# Brain Metastases from Ovarian Cancer: Current Evidence in Diagnosis, Treatment, and Prognosis

**DOI:** 10.3390/cancers12082156

**Published:** 2020-08-04

**Authors:** Fulvio Borella, Luca Bertero, Antonio Morrone, Alessandro Gambella, Marialuisa Bovetti, Stefano Cosma, Andrea Carosso, Dionyssios Katsaros, Silvia Gemmiti, Mario Preti, Giorgio Valabrega, Giulia Scotto, Paola Cassoni, Chiara Benedetto

**Affiliations:** 1 Department of Surgical Sciences, University of Turin, 10126 Turin, Italy; fulvio.borella@unito.it (F.B.); marialuisa.bovetti@unito.it (M.B.); stefano.cosma@unito.it (S.C.); andrearoberto.carosso@unito.it (A.C.); dionyssios.katsaros@unito.it (D.K.); silvia.gemmiti@edu.unito.it (S.G.); mario.preti@unito.it (M.P.); chiara.benedetto@unito.it (C.B.); 2 Pathology Unit, Department of Medical Sciences, University of Turin, 10126 Turin, Italy; morant592@libero.it (A.M.); alessandro.gambella@unito.it (A.G.); paola.cassoni@unito.it (P.C.); 3 Obstetrics and Gynecology Unit 1, Sant’ Anna Hospital, University of Turin, 10126 Turin, Italy; 4 Candiolo Cancer Institute, FPO-IRCCS, Strada Provinciale 142 Km 3.95, Candiolo, 10060 Torino, Italy; giorgio.valabrega@unito.it (G.V.); giulia.scotto@ircc.it (G.S.); 5 Department of Oncology, University of Turin, Candiolo, 10060 Torino, Italy

**Keywords:** brain metastases, ovarian cancer, BRCA, treatment, management, pathology, diagnosis, radiotherapy, surgery

## Abstract

With this review, we provide the state of the art concerning brain metastases (BMs) from ovarian cancer (OC), a rare condition. Clinical, pathological, and molecular features, treatment options, and future perspectives are comprehensively discussed. Overall, a diagnosis of high-grade serous OC and an advanced disease stage are common features among patients who develop brain metastases. *BRCA1* and *BRCA2* gene mutations, as well as the expression of androgen receptors in the primary tumor, are emerging risk and prognostic factors which could allow one to identify categories of patients at greater risk of BMs, who could benefit from a tailored follow-up. Based on present data, a multidisciplinary approach combining surgery, radiotherapy, and chemotherapy seem to be the best approach for patients with good performance status, although the median overall survival (<1 year) remains largely disappointing. Hopefully, novel therapeutic avenues are being explored, like PARP inhibitors and immunotherapy, based on our improved knowledge regarding tumor biology, but further investigation is warranted.

## 1. Introduction

Ovarian cancer (OC) is the current leading cause of gynecological cancer deaths. In the United States, over 20,000 new diagnoses of OC were estimated for 2020, and over 13,000 deaths due to this tumor type [1]. OC is characterized by a high frequency of loco-regional recurrence caused by the dissemination of neoplastic cells through peritoneal fluid or lymphatic drainage [2,3,4,5]. Despite sensitivity to chemotherapy, most tumor relapses are observed within three years from the end of adjuvant treatments [6]. The most common metastatic sites are peritoneum and omentum (86%), pelvic and/or para-aortic lymph nodes (70%), bowel (50%), and spleen (20%) [5].

Brain metastases (BMs) represent the most common adult intracranial malignancy, and it is estimated that approximately 20–30% of patients with a solid tumor will develop this complication during the disease course; moreover, data from autopsies of patients with malignant tumors showed an even higher incidence (up to 40%), suggesting that BMs can be frequently undetected [7,8]. Breast, lung, colorectal cancers, and melanoma are the neoplasms most frequently associated with the development of BMs [7,8,9,10], while the central nervous system (CNS) seeding from OC is more rarely observed. Within the last 30 years, excluding single case reports or small series (*n* < 10), only the outcomes of approximately 1100 patients have been described (Table 1) [11,12,13,14,15,16,17,18,19,20,21,22,23,24,25,26,27,28,29,30,31,32,33,34,35,36,37,38,39,40,41,42,43,44,45,46,47,48]. OC cells can reach the CNS through the bloodstream or, more rarely, they may invade the meninges through direct invasion from a bone metastasis, or by lymphatic drainage [49]. The incidence of BMs in OC is estimated at 1.34%, ranging from 0.49% to 6.1% (Table 1) of cases. This heterogeneity can be partially explained by improvements in diagnostic procedures and treatments, which improved detection and outcome rates. Despite the available therapeutic options, such as surgery, radiotherapy, and chemotherapy, there are no established guidelines for the management of this severe complication and prognosis remains poor [5,49,50,51], with a median overall survival of 10.1 months (Table 1). The growing incidence of BMs from OC warrants specific attention and focused research as prompt treatment may influence the prognosis.

The purpose of this review is to provide a comprehensive and up to date overview of risk and prognostic factors, clinical and pathological features, available treatments, and future therapeutic options for BMs from OC.

## 2. Risk and Prognostic Factors

### 2.1. Clinical Characteristics

Based on literature data, most patients who developed BMs had a high-grade serous OC as primary tumor (79%), followed by the endometrioid, mucinous and clear cell histotypes. Nevertheless, it should be noted that BMs can also occur in low-grade primary neoplasms (Table 1). Furthermore, 86% of patients had an advanced FIGO stage (III–IV) at OC diagnosis (Table 1).

Despite the rarity of BMs from OC, many prognostic factors have been identified. Patients who were <50 years at the primary tumor time of diagnosis [50] and with a Karnofsky performance status (KPS) ≥70 were associated with a better prognosis [35,37,44,49,50], while the number of previous extracranial recurrences before the diagnosis of BMs and the number of brain lesions (more than one) was reported as a significant unfavorable prognostic factor by several authors [30,33,35,40,45,49,50]. The prognostic significance of concomitant extracranial disease is controversial. Paknenshan et al. [50] found no relationship with survival, while Marchetti et al. [40] reported a worse survival rate. Similarly, the platinum sensitivity of the primary tumor has been linked to a better prognosis by Sehouli et al. [30] (HR 0.23, 95% CI 0.12–0.48), but this finding has not been confirmed by other authors [40,46]. The specific OC histotype, CA-125 levels and FIGO tumor stage at primary diagnosis and the residual tumor after surgery do not seem to affect the prognosis [35,50].

### 2.2. Hormone Receptors

Unfortunately, the implications of hormone [estrogen (ER), progesterone (PgR) and, androgen (AR)] receptors expression on the risk and prognosis of BMs from OC have been poorly investigated. Mittica et al. [42] compared the hormone receptors expression of 11 OC and their matched BMs with a control series of 22 OCs without brain involvement: BMs showed lower expression of ER and AR compared to the corresponding primary tumor. Furthermore, they also observed that the absence of AR expression in OC carries a 9.5-fold increased risk to develop BMs compared with AR-positive OC. This relationship between AR expression and risk of developing BMs has been confirmed by a second study of the same group [48], in an independent validation set of 19 new OC. In addition, a significantly worse survival outcome, both in terms of PFS (*p* = 0.005) and brain-specific PFS (*p* = 0.002), was observed in patients with low expression of AR (<10%). The group also investigated the role of HER-2 expression, but found no relationship with the risk of developing BMs or prognosis.

Despite the overall limitations in terms of available data, some information could be gathered from breast cancer, since this topic has been extensively investigated in this tumor type. A recent meta-analysis [52] suggested that ER negativity was an independent risk factor for BMs development, and that the triple-negative (ER, PgR and HER-2 negative) breast cancers showed shorter time to BMs development from the diagnosis of the primary tumor, compared to other breast cancer subtypes. Few data are available concerning AR expression, but overall it appears that this receptor is not related to the risk of developing distant metastases in triple-negative breast cancer [53]. Similar research efforts are warranted also for OC, to fully elucidate the role of hormone receptors as potential risk factors for BMs development.

### 2.3. BRCA Status

Germline mutations in the homologous DNA repair genes *BRCA1* and *BRCA2* account for about 5–10% of all breast cancers and for 10–18% of all OCs [54]. In particular, women who carry a *BRCA1* or *BRCA2* mutation have a cumulative lifetime risk of developing OC of 39–54% and 11–23%, respectively [55]. Furthermore, The Cancer Genome Atlas (TCGA) studies have shown that approximately half of the cases of serous OC harbor homologous recombination deficiency (HRD) [56]. Regarding the prognostic significance of HRD in OC, *BRCA*-deficient OC patients show higher survival rates and are more responsive to platinum-based chemotherapy [55,57,58,59]. A recent systematic review with meta-analysis [60] showed a benefit in PFS and overall survival (OS) for both *BRCA1* (PFS HR: 0.68, 95% CI: 0.52–0.89; OS HR: 0.73, 95% CI: 0.63–0.86) and *BRCA2* (PFS HR: 0.48, 95% CI: 0.30–0.75; OS HR: 0.57, 95% CI: 0.45–0.73) mutations’ carriers, compared with *BRCA* wild type patients. On the other hand, *BRCA*-mutated OC has a greater predisposition to develop visceral metastases outside the pelvis than non-mutated tumors [61]. A potential relationship between a positive family history for hereditary breast and OC and the risk of developing BMs in OC patients has been suggested by Jernigan et al. [62] However, few authors have extensively evaluated the role of HRD in BMs from OC. Ratner et al. [63], analyzing a cohort of 4515 OCs (473 *BRCA* mutated and 1679 *BRCA* wild type), found 46 patients who developed BMs (1%). Among the *BRCA* mutated patients, 3% (14/473) developed BMs, while within the *BRCA* wild type group, the BMs rate was 0.6% (10/1679). Moreover, *BRCA* mutated patients who developed BM had a lower mean age at primary tumor diagnosis (60 years vs. 63.5 years). The estimated HR for developing BMs in *BRCA* mutation carriers was 3.84 (95% CI: 1.60–9.22, *p* < 0.001), but no difference was observed between patients with or without *BRCA* mutations in terms of survival.

A recent study [64] explored the characteristics of 96 OC women who developed BMs, according to *BRCA* status (21 *BRCA* mutations carriers and 63 *BRCA* wild type patients). *BRCA* mutations carriers showed a better OS (29 months vs. 9 months), with an HR of 0.53 after stratifying for the presence of systemic disease (95% CI: 0.25–1.11, *p* = 0.09). The longer disease course of HRD patients may explain the higher predisposition to develop extra-abdominal disease, although in the previously reported study [63], patients with *BRCA* mutation developed BMs on average 8 months earlier than the *BRCA* wild type (median time 27 months versus 35 months) therefore, this assumption should be furtherly verified and the longer course of the disease in mutated patients may not be a relevant factor for the development of BMs.

## 3. Clinical Presentation and Diagnosis

The clinical presentation of BMs is variable, and depends on the location and the number of metastases. Overall, mild or severe headache is one of the most common symptoms and is present in up to 50% of cases. This finding typically occurs in patients with multiple metastases or with posterior fossa lesions, and can also be associated with papilledema (15–25%). Up to 40% of patients with BMs showed focal neurological deficits, while seizures occurred in 15–20% of cases. Confusion is also a common symptom, especially in patients with multiple metastases and/or in the case of intracranial hypertension [9,65].

Diagnosis of BMs from OC before or synchronous with the primary tumor is an extremely rare event [50], thus most diagnoses occur during the disease course. At presentation, most of the cases showed multiple metastatic lesions (58.1%) (Table 1), and were symptomatic at the time of BMs diagnosis. However, in one of the largest series reported so far, no symptoms were observed at diagnosis in about 26% of cases, [40] therefore, the diagnosis can also be incidental in a significant subgroup of patients. For this reason, some authors [40,48] have proposed to establish an active surveillance protocol aimed at high risk patients, as early detection may enable improved outcomes. The most commonly reported symptoms in BMs secondary to OC are headache, motor deficit/weakness, seizures, nausea and vomiting, dysphasia/aphasia, and vertigo (Table 1). The anatomical sites most frequently affected include cerebellum (30%) and frontal (20%), parietal (18%), and occipital (11%) lobes [50].

In patients with a history of OC, BMs can be suspected by consistent clinical and radiological (computed tomography—CT and/or magnetic resonance imaging—MRI) findings. For imaging, brain MRI is the technique of choice, because it has a higher resolution and allows one to study the posterior fossa and the presence of leptomeningeal disease more accurately than CT [9,26,66]. BMs from OC can also be occasionally detected by Positron emission tomography (PET)/PET-CT [67], but this technique does not provide a spatial resolution comparable with MRI.

Regarding circulating biomarkers, the role of CA-125 in the diagnosis and management of BMs from OC is unclear. CA-125, at the time of diagnosis of BMs, was positive in 58.5% of cases, but this marker is obviously affected by systemic disease status, and many patients with BMs also had an active extra-cranial disease, thus its real relevance to detect BMs cannot be inferred. Furthermore, CA-125 values were not found to be related to the time interval between the primary OC and the diagnosis of BMs. Other biomarkers, such as CA 72-4, lactate dehydrogenase (LDH), epithelial membrane antigen (EMA), chromogranin, CD-56, pan-CK, along with human chorionic gonadotropin (HCG) and alpha fetoprotein (AFP), have been rarely studied, and no correlation with BMs has been reported [50]. Finally, data on the role of human epidydimal protein 4 (HE4) are not available.

## 4. Pathology and Molecular Profiling

**Figure 1:**
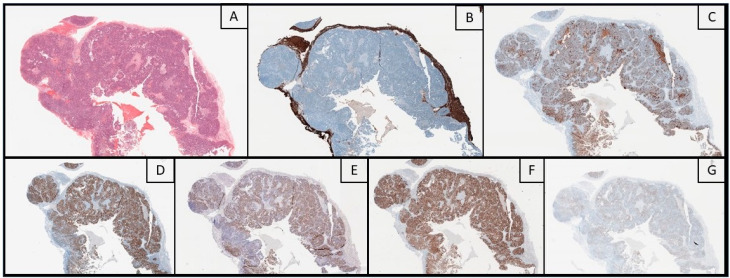
Histological images (original magnification: 20**X**) of a brain metastasis from ovarian cancer. Hematoxylin-eosin stain (**A**) shows a papillary carcinoma with hyperchromic nuclei and prominent nucleoli, surrounded by GFAP-positive brain parenchyma (**B**). Immunohistochemistry showed a consistent profile: positive stainings for CA-125 (**C**), Estrogen Receptors (**D**), Wilms Tumor-1 (**E**) and PAX-8 (**F**) and, focally, for Androgen Receptors (**G**).

Histology is usually consistent with the primary tumor. In BMs from serous high-grade serous OC (HGSC), markedly atypical epithelial cells with pleomorphic hyperchromatic nuclei and prominent nucleoli can be observed within a papillary architecture [68], but, despite the presence of specific histopathological features, reaching a conclusive diagnosis can be challenging. For instance, in patients with multiple primary tumors, or if the primary OC is still undiagnosed, a careful evaluation is warranted, since morphological patterns and immunohistochemical (IHC) markers can overlap with other tumor entities. Nafisi et al. proposed a 6-step diagnostic flowchart, taking also into account the differential diagnosis between HGSC, clear cell (CCC) and endometrioid (EC) carcinoma. Other than clinical history, IHC positivity for CK7, ER and, paired box 8 (PAX-8) matched with negativity for CK20, support the Mullerian origin of the tumor. This pattern is reliable for any type of ovarian metastasis, although the expression of the single markers can vary among the different histotypes. Strong and diffuse positivity for Wilms tumor 1 (WT1), together with high expression of p53 and p16, point towards HGSC; conversely, WT1 negativity represents a clue for other tumor entities like CCC and EC, which are usually ER-negative and positive, respectively [69]. Moreover, it should be stressed that correlation with clinical and radiological findings is always extremely important to avoid diagnostic pitfalls, especially in patients without a previous oncological history. An example of histological features of BMs from OC is shown in Figure 1.

Regarding molecular profiling, Masoodi et al. evaluated the primary tumor and matched metastases of six patients with OC, showing branching evolution patterns among the primary tumor cells of high-grade serous OC, whereas linear and parallel metastatic progression was observed in 67.3% and 33.7% of cases, respectively [70]. Increased single nucleotide variations and loss of heterozygosity events and more extensive copy-number alterations were also reported in metastatic disease [70,71]. Concerning specific genes, *BRCA1* resulted in being the most commonly altered gene in OC BMs [72]. A next-generation sequencing-based genomic analysis of eight OC BMs exhibited *BRCA1/2* mutations in 7/8 cases, and all samples showed mutations in at least one gene involved in DNA repair (*BRCA1/2*, *ATM*, *CHEK2*) [72]. Furthermore, two authors demonstrated a high prevalence of *BRCA1* protein loss in patients with BMs from OC [73,74].

Differences have been observed between primary tumors and metastatic lesions [75] in terms of transcriptomic profiles, but conflicting data have also been reported [76]. Regarding specific markers, Matsuo et al. found a higher expression of *MDR1* in primitive ovarian cancers which then developed BMs and in the BMs themselves; this finding has therapeutic relevance, since a higher *MDR1* expression is associated with resistance to chemotherapy drugs like paclitaxel [77]. Choi et al. reported a higher PD-L1 expression in brain metastasis compared to primary tumors [71] with potential implications for immunotherapy [78]. Indeed, the immune system seems to play a significant role in shaping OC metastasis development: a higher expression of *IL7R*, probably related to T cells activation and infiltration, was found in OC metastases, as well as differences in *CALB2*, *CYP1B1*, *EFTUD1*, *RARRES2* and *TIMP3* expressions [79]. Taken together, these genes have been suggested as a potential signature to distinguish metastatic lesions from primary cancers, as well as a potential tool to predict the metastatic risk. Furthermore, a higher expression of *MYC*, *IRF1*, *BCL2L2*, *TNFSF10* was reported in metastasis, suggesting an anti-apoptotic and proliferative behavior. *AXIN2*, *DKK2*, *NKD1/2* also resulted in being highly expressed [79], supporting the WNT-β-catenin pathway activation [80,81], and similar results were reported for the JAK-STAT and NOTCH pathways [79].

Finally, as previously anticipated, BMs show a reduction in the expression of hormonal receptors compared to the matched primary tumors, in particular PgR and AR. This finding suggests a potential "dedifferentiation" of neoplastic cells during the BMs development [42].

Proteomic analyses could also be exploited to identify novel potential therapeutic targets for OC BMs. For instance, Yoshida et al. identified a wide range of differentially expressed proteins between OC primary tumor and BMs. Among these, a strong signal was found for alpha-enolase, triosephosphate isomerase and transgelin-2 [82].

Lastly, considering the growing importance of extensive molecular profiling to tailor patients’ treatments, liquid biopsy approaches could be applied to overcome the limitations in sampling CNS lesions. Although the analysis of circulating tumor DNA (ctDNA) or of other nucleic acids it is not commonly used in clinical practice for sampling BMs from OC, multiple reports [83,84] showed the possibility to gather informative data in multiple types of primary and secondary CNS tumors; thus, this possibility could be investigated in future studies targeting OC BMs.

## 5. Treatments

### 5.1. Radiotherapy

Radiotherapy is one of the main treatment options for BMs. Whole brain radiotherapy (WBRT) was considered as the best approach for many years, since it allows one to target both macro- and microscopic lesions, allowing one to achieve good local disease control [9,85]. Nowadays, WBRT can still represent the first option for the treatment of BMs from OC in the presence of multiple lesions, with or without extra-cranial disease [50]. A recent study on 21 patients showed that WBRT alone had a positive effect on survival compared to regimens that did not include WBRT: 45 months (95% CI 35–54.9) versus 19 months (95% CI 11.1–26.8,) (*p* < 0.001), with an HR of 0.152 (95% CI 0.033–0.695, *p* = 0.015) [47]. In a large series of 72 patients, Cohen et al. [22] reported that WBRT in combination with surgery showed a more favorable survival (median 23 months) than WBRT (median 5.33 months) or surgery (median 6.9 months) alone (*p* < 0.01). However, WBRT is burdened by significant side effects, such as fatigue, somnolence, and memory impairments, and a literature review estimated a poor median survival with exclusive WBRT (3–6 months, range 1.5–27) [66].

Gamma knife radio surgery (GKRS) is an alternative option that provides satisfactory results, especially in patients with single metastases, severe comorbidity, or who cannot undergo surgical resection. Lee et al. [28] compared the outcomes of 7 patients treated with GKRS with 8 patients treated with WBRT as the primary treatment modality. GKRS-treated patients showed a better survival (median, 29 months) compared to WBRT (median, 6 months) (*p* = 0.00061), regardless of the number of metastases. Superior survival outcomes in patients treated with GKRS were also reported in a number of small retrospective series [26,34].

### 5.2. Surgery

Surgical excision of BMs can be considered as an alternative to radiotherapy and may be useful in selected patients, to control intracranial hypertension, symptoms, and improve survival. Furthermore, brain surgery allows one to obtain tissue for histopathological analysis and for the identification of prognostic and predictive markers [9].

Several authors reported better results in terms of local control and survival compared with chemotherapy and radiotherapy alone [20,25,49,50,86]. A literature review showed that surgery combined with radiotherapy +/− chemotherapy resulted in better outcomes (median OS 21.8 and 20.15 months) compared with surgery (6.5 months) or radiotherapy alone (5.4 months) [66].

More recently, a case series of 12 patients including an analysis of 20 studies conducted by Niu et al. [36] reported higher survival rates in patients treated with GKRS plus surgical excision, compared to those who did not receive this treatment (25 months vs. 6 months, *p* < 0.001).

Overall, patients with good general conditions (KPS > 70), a single BM and no extra-CNS localizations seem to be the best candidates for surgical therapy [9,20,87], but selected patients with extra-cranial disease or multiple brain metastasis can also benefit from surgery in terms of symptoms and quality of life improvements [66].

### 5.3. Chemotherapy with Cytotoxic Agents

Despite the significant advancements achieved in the treatment of solid malignancies and the advent of the precision medicine era, the cornerstone of neoadjuvant and adjuvant treatment of OC remains the intravenous administration of carboplatin plus paclitaxel every 3 weeks [88,89]. Regarding recurrent disease, in case of a ≥ 6 months therapy-free interval as defined by the Gynecologic Cancer Intergroup [90], a platinum-based re-challenge therapy can be proposed, or, in the case of platinum-refractory relapses, other cytotoxic agents such as pegylated liposomal doxorubicin (PLD), topotecan, gemcitabine, trabectedin or weekly paclitaxel, eventually with the addition of an anti-angiogenetic drug, can be considered [89,91,92].

The role of chemotherapy with cytotoxic agents in BMs treatment is controversial. Indeed, the major limitation to its use is linked to the difficulty of many drugs to cross the blood-brain barrier (BBB) and reach adequate therapeutic concentrations within the CNS. Furthermore, the addition of chemotherapy to WBRT did not always provide survival benefits in trials investigating this issue [93].

Platinum-based chemotherapy (carboplatin and cisplatin) represents the cornerstone of OC medical treatment and can be used in both primary and secondary brain tumors thanks to its ability to cross the BBB [94,95]. However, data about the role of chemotherapy on BMs from OC are scant. Some BMs remissions have been achieved in patients undergoing platinum-based regimen, with or without docetaxel, but these results remain anecdotal [15,96,97,98,99]. In addition, in patients treated with exclusive systemic chemotherapy, the median survival remains poor (2.5 to 7 months) [100]. As mentioned above, the prognostic role for BMs of previous platinum sensitivity is not yet defined, however, this aspect must be considered when attempting a re-challenge with platinum-based chemotherapy.

If platinum-based chemotherapy is not possible, other drugs capable of crossing the BBB, such as topotecan [101] and gemcitabine [102] (high concentration in the brain was reported, particularly after radiotherapy for glioblastoma or in combination with other chemotherapeutic agents), can be considered. Regarding anthracyclines, the glutathione PLD can reach a brain-to-blood ratio 4.8-fold higher compared to the uncoated PLD [103]. Conversely, the ability of trabectedin to cross the BBB is currently unknown.

Several new techniques are being proposed to increase the permeability of BBB and to selectively kill OC cells without affecting the surrounding healthy tissues: similar approaches could also be experimented to optimize treatment of BMs [104,105,106].

### 5.4. Trimodal Approach

Considering the overall advantages and disadvantages of the available treatment option, it should be noted that the best survival results for BMs from OC have been obtained with a trimodal therapy (radiotherapy, surgery, and chemotherapy), while monotherapy is associated with poor survival (HR: 2.57, 95% CI: 1.64–3.86) [40]. Pakneshan et al. [50] reported a significant improvement in survival (median OS 20.5 months) in patients treated with this approach. Similar results were observed by Anupol et al. [16] (median OS 20.5 months), Chiang et al. [33] (median OS 30 months), and Marchetti et al. [40] (median OS 24 months).

### 5.5. PARP Inhibitors

Poly (ADP) ribose polymerase (PARP) is a family of nuclear proteins involved in the repair of single-strand DNA breaks through a process known as PARylation. In particular, PARP-1 is the most common PARP isoform, and in the majority of tissues it accounts for about 90% of total cellular PARP activity [107,108]. Several inhibitors of the poly ADP ribose polymerase (PARPi) have allowed an increase in OC PFS, and have been approved by the Food and Drugs Administration (FDA) for treatment maintenance after adjuvant chemotherapy and platinum-sensitive recurrence. Such inhibitors include Olaparib for *BRCA* mutation carriers, Niraparib and Rucaparib for OC regardless the *BRCA* status [109,110,111,112]. PARPi have the ability to cross the BBB [113,114,115], and are currently under investigation for the treatment of BMs in triple-negative breast cancer [116,117]. PARPi could also play a role in the treatment of BMs from OC, and, despite the current lack of data in a large series of patients, three cases have been reported: 

- a 61-year-old woman with *BRCA2* mutation and diffuse leptomeningeal disease from a high-grade serous OC showed a good clinical response associated with improved symptoms and quality of life, after been treated with Olaparib. Disease progression was observed 12 months after starting the PARPi treatment [118].

- a 58-year-old woman with *BRCA1* mutation and multiple BMs from high-grade serous OC achieved a complete response after a 21-month treatment with Olaparib [119].

- a 68-year-old woman with BM from high-grade serous OC was treated with Niraparib as maintenance therapy and remained free of disease progression for more than 17 months [120].

Although these cases remain anecdotal, the results are encouraging, and there is a strong preclinical rationale supporting the use of PARPi also in these patients, especially in *BRCA* mutated tumors.

### 5.6. Immunotherapy

It is well-known that the interactions between the tumor and the immune system play a fundamental role in cancer progression as well as treatment. In fact, the inhibition of T cell checkpoint molecules such as programmed cell death protein 1 (PD-1), its ligand (PD-L1) and cytotoxic T-lymphocyte antigen 4 (CTLA-4) using monoclonal antibodies (immune checkpoint inhibitors (ICIs)) has changed the natural history of multiple solid malignancies, including melanoma, non-small cell lung cancer (NSCLC), and renal cell cancer [121]. However, initial data on the use of ICIs for OC treatment have shown disappointing results, with median response rates of 10–15% [122,123,124,125,126,127]. A study on NSCLC derived BMs suggested a higher expression of PD-L1 compared with the matched primary tumor [128]. Furthermore, a work of the same group investigating the role of tumor-infiltrating lymphocytes (TILs) in a series of BMs derived from melanoma, lung, breast and renal cancer showed that the presence of dense TILs infiltrates is common and related with survival [129]. These findings suggest a role of immunotherapy in the treatment of BMs [130]. Currently, the immunological profile of BMs from OC has been investigated in 2 cases only, and, as previously mentioned, these brain lesions showed a high mutational burden and increased PD-L1 expression compared with primary tumors [71]. In fact, some data suggested a role of features like the clear cell histotype, PD-L1 expression by cancer cells, *BRCA* status, microsatellite instability, and tumor mutational burden in predicting OC response to immunotherapy [131]. These markers could help to identify patients who might benefit from immunotherapy and thus be evaluated in future studies focused on OC BMs. Finally, there is a strong rationale supporting the use of radiotherapy to enhance the response to immune checkpoint inhibitors in OC treatment [132]. This combination showed synergism in the treatment of small series of patients affected by BMs from melanoma [133,134], suggesting a potential therapeutic efficacy also for brain lesions from OC.

## 6. Conclusions

BMs from OC remain a rare event, and the overall quality of current evidence is limited, since it is mainly based on heterogeneous retrospective series. 

No specific follow-up strategy for early identification of BMs is currently recommended in patients with a history of OC, although, as mentioned above, up to 26% may be asymptomatic at diagnosis. In this context, women with *BRCA* mutation and low AR expression in primary cancer could benefit from a targeted follow-up, as they are at greater risk of developing BMs, but prospective studies are warranted to establish reliable risk and prognostic factors for OC BMs.

Concerning treatments, a multimodal strategy (including surgery, radiotherapy and chemotherapy), if feasible, seems to be the best way to improve survival rates compared to other therapeutic approaches. Novel avenues are becoming available based PARPi and immunotherapy, but further studies are needed.

## Figures and Tables

**Table 1:** Clinico-pathological features, treatments and outcome data of patients with BMs from OC. Only series published since 1990 and with at least 10 cases were reported.

Author	BM (N)	Incidence	Histotype (N, %)	Grade (N, %)	FIGO Stage at OC Diagnosis (N, %)	Median Age at OC Diagnosis (yrs)	Mean Interval to BMs (mo)	Single vs. Multiple BMs (N, %)	Most Common Symptoms (N, %)	Treatments	Median Survival (mo) from the Diagnosis of BM
LeRoux [11] 1990	14	1.1%	Serous (7, 50%) ED (2, 14%) CC (1, 7%) MC (2, 14%) Other (2, 14%)	G1 (1, 7%) G2 (9, 64%) G3 (4, 28%)	I (3, 21%) II (4, 29%) III (4, 29%) IV (3, (21%)	52.5	14.5	9 (64%) vs. 5 (36%)	Motor Weakness (5, 36%) Seizure (5, 36%) Headache (2, 14%) Confusion (1, 7%) Speech disturbance (1, 7%)	RT (8, 57%) Surg + RT (5, 36%) No treatment (1, 7%)	3
Rodriguez [12] 1992	15	1.9%	Serous (13, 87%) MC (1, 6.7%) Other (1, 6.7%)	G1 (1, 6.7%) G2 (3, 20%) G3 (8, 53.3%) NA (3, 20%)	I (2, 13.3%) III (8, 53%) IV (5, 33%)	54.5	18.5	5 (33%) vs. 10 (67%)	Headache (6, 40%) Seizure (3, 20%) Aphasia (1, 6.7%) Decreased arm coordination (1, 6.7%) Vertigo (2, 13.3%) Personality change (1, 6.7%) Confusion (1, 6.7%)	RT + CHT (6, 40%) RT (4, 26.7%) Surg + RT + CHT (3, 20%) Surg + RT (1, 6.7%)	9
Geisler [13] 1995	16	3.3%	Serous (16, 100%)	G1 (1, 6.25%) G2 (6, 37.5%) G3 (9, 56.25%)	III (12, 75%) IV (4, 25%)	56.6	19	8 (50%) vs. 8 (50%)	Headache (5, 31.2%) Seizure (2, 12.5% Vertigo (2, 12.5%) Paresis (2, 12.5%) Atassia (2, 12.5%) Aphasia (1, 6.25%) Syncope (1, 6.25%) Confusion (1, 6.25%)	RT (8, 50%) Surg + RT (5, 31.25%) RT + CHT (2, 12.5%) No treatment (1, 6.25%)	3
Corn [14] 1995	32	0.8%	NA	NA	NA	56	24	13 (40%) vs. 17 (53%) NA 2 (6%)	Headache (12, 37,5%) Seizures (5, 16%)	RT (27, 84%) RT + CHT (5, 16%)	4
Cormio [15] 1995	23	NA	Serous (14, 61%) ED (6, 26%) CC (1, 4%) Other (2, 9%)	G1 (2, 9%) G2 (4, 17%) G3 (17, 74%)	I (3, 13%) III (17, 74%) IV (3, 13%)	56	35	9 (41%) vs. 13 (59%)	NA	RT (14, 60.9%) Surg + RT (5, 21.7%) NA (4, 17.4)	5
Anupol [16] 2002	15	1.4 %	Serous (14, 93.3%) Other (1, 6.7%)	G2 (1, 6.7%), G3 (12, 80%), NA (2, 13.3%)	III (7, 47%) IV (8, 53%)	58	22	7 (46.7%) vs. 7 (46.7%) NA: 1 (6.6%)	Decreased mental status (7, 46%) Motor deficit (4, 20%) Headache (3, 20%) Visual disturbance (1, 6%) Seizure (1, 6%)	RT + CHT + Surg (5, 33,3%) RT + CHT (4, 26,7%) RT (3, 20%) No treatment (2, 13,3%) RT + Surg (1, 6.7%)	6
Pothuri [17] 2002	14	NA	Serous (9, 64%) ED (2, 14%) Other (3, 21%)	G2 (4, 29%) G3 (10, 71%)	II (1, 7%) III (12, 86%) IV (1, 7%)	NA	42	2 (14%) vs. 12 (86%)	Headache (6, 43%) Hemiparesis (4, 29%)	Surg + CHT + RT (6, 43%) Surg + RT (5, 36%) Surg + CHT (2, 14%) Surg (1, 7%)	18
Sanderson [18] 2002	13	1.1%	Serous (3, 23%) CC (2, 15.4%) ED (1, 7.7%) Other (7, 53.8%)	NA	III (6, 46.1%) IV (7, 53.9%)	55	36		NA	RT (7, 53.8%) Surg (3, 23.1%) CHT (2, 15.4%) No treatment (1, 7.7%)	4
Kolomainen [19] 2002	18	0.49%	Serous (13, 72%) MC (1, 5.5%) ED (1, 5.5%) Other (3, 17%)	G2 (8, 44%) G3 (9, 50%) NA (1, 6%)	I (6, 33%) II (3, 17%) III (7, 39%) IV (2, 11%)	52	46	9 (50%) vs. 9 (50%)	Motor weakness (6, 33.3%) Confusion (6, 33.3%) Headache (5, 27.8%) Seizures (3, 16.7%)	CHT (18, 100%)	7
Cormio 2003 [20]	22	NA	Serous (15, 68%) ED (5, 22%) CC (1, 5%) Other (1, 5%)	G1 (4, 17%) G2 (6, 28%) G3 (12, 55%)	I (2, 8%) III (19, 88%) IV (1, 4%)	54	29	NA	NA	Surg + RT (17, 77%) Surg+ CHT (5, 23%)	16
Kumar [21] 2003	18	2.7%	Serous (13, 72%) MC (2, 11%) ED (1, 6%) Other (2, 11%)	G1 (1, 6%) G2 (5, 28%) G3 (11, 61%) NA (1, 6%)	I (1, 6%) III (13, 72%) IV (4, 22%)	54	29	5 (28%) vs. 13 (72&%)	Headache (10, 56%) Weakness (9, 50%) Seizure (3, 17%) Vision problems (2, 11%) Tremors (2, 11%) Vertigo (2, 11%) Aphasia (2, 11%) Altered Consciousness (1, 6%)	RT (8, 44%) CHT + RT (5, 28%) Surg + RT + CHT (4, 22%) No treatment (1, 6%)	4
Cohen [22] 2004	72	0.9%	Serous (14, 24%) MC (6, 10%) CC (2, 3%) ED (2, 3%) Other (35, 48%) NA (13, 18%)	G1-G2 (11, 15%) G3 (52, 72%) NA (9, 13%)	I–II (12, 16%) III–IV (52, 72%) NA (8, 11%)	50.4	22	25 (35%) vs. 47 (65%)	Neurological deficit (41, 57%) Headache (18, 25%) Seizures (11, 15%) Dizziness (4, 6%)	RT (36, 50%) Surg + RT (13, 18%) Surg (8, 12%) Steroids (8, 12%) CHT (3, 4%) NA (3, 4%) RT + CHT (1, 1%)	6
Pectasides [23] 2005	17	1.17%	Serous (12, 71%) MC (2, 12%) ED (2, 12%) Others (1, 6%)	G2 (3, 18%) G3 (11, 65%) NA (3, 18%)	II (2, 12%) III (12, 71%) IV (3, 18%)	56	16	5 (29%) vs. 12 (71%)	NA	RT + CHT (6, 35%) No treatment (5, 29%) RT (4, 24%) Surg + CHT + RT (2, 12%)	5.7
D’Andrea [24] 2005	11	NA	Serous (11, 100%)	NA	II (11, 100%)	60.3	21	11 (100%) vs. 0 (0%)	Hemiparesis (8, 73%)	Surg + RT + CT (11, 100%)	28
Chen [25] 2005	19	NA	Serous (7, 37%) CC (1, 5%) ED (1, 5%) Other (10, 53%)	G3 (13, 68%) NA (6, 32%)	I (1, 5%) III (13, 68%) IV (5, 26%)	51	25	7 (37%) vs. 12 (63%)	Headache (12, 63%) Motor deficit (7, 37%) Mental status change (4, 21%) Seizure (3, 16%) Ataxia (2, 11%) Memory (3, 16%) Sensory deficit (1, 5%) Aphasia (1, 5%)	RT (10, 53%) Surg + RT (8, 42 %) Surg (1, 5%)	16
Kim [26] 2007	13	2.7%	Serous (9, 69%) MC (1, 8%) ED (1, 8%) CC (1, 8%) Other (1, 8%)	G1 (1, 8%) G2 (1, 8%) G3 (7, 54%) NA (4, 30%)	I (1, 8%) III (9, 69%) IV (3, 23%)	52	28	2 (15%) vs. 11 (84%)	Headache (9, 69%) Seizure (2, 15%) Weakness (2, 15%)	RT (7, 54%) RT + CHT (4, 30%) RT + Surg (1, 8%) No treatment (1, 8%)	7
Gadducci [27] 2007	12	6.1%	Serous (8, 67%) ED (1, 8%) CC (1, 8%) Other (2, 17%)	G2 (6, 50%) G3 (6, 50%)	III (9, 75%) IV (3, 25%)	59.5	33.5	6 (50%) vs. 6 (50%)	Headache (4, 33%) Weakness (3, 25%) Seizure (1, 8%) Impaired deambulation (3, 25%)	RT (5, 42%) RT + CHT (3, 25%) No treatment (2, 17%) Surg (1, 8%) Surg + RT (1, 8%)	8.3
Lee [28] 2008	15	1.1%	Serous (8, 53%) ED (3, 20%) MC (1, 7%) Other (3, 20%)	NA	I (1, 7%) III (9, 60%) IV (5, 33%)	55	28	5 (33%) vs. 10 (67%)	Headache (6, 40%) Loss of consciousness (1, 7%) Motor weakness (4, 27%) Nausea and vomiting (5, 15%) Hemiparesis (1, 7%) Atassia (1, 7%)	RT + CHT (9, 60%) RT (6, 40%)	14
Ratner [29] 2009	24	1.1%	Serous (17, 71%) ED (3, 12%) Other (4, 17%)	G2 (3, 12%) G3 (21, 88%)	I (1, 4%) III (14, 58%) IV (5, 21%) NA (4, 17%)	56	23	7 (29%) vs. 17 (71%)	Mental status change (4, 17%) Seizures (3, 13%) Hemiparesis (3, 13%) Dysphasia (3, 13%) Cerebrovascular accident (2, 8%) Diplopia (2, 8%) Headache (2, 8%) Gait disturbance (2, 8%) Neuropathy and incontinence (1, 4%) Loss peripheral visual field (1, 4% )Twitching hands (1, 4%)	RT (18, 75%) Surge + RT (3, 13%) No treatment (2, 8%) Surg (1, 4%)	8.5
Sehouli [30] 2010	74	1.8%	Serous (53, 72%) Other (21, 28%)	G1-G2 (31, 42%) G3 (43, 58%)	I/II (12, 16%) III/IV (62, 84%)	53.9	28.8	26 (35%) vs. 48 (65%)	Headache (25, 34%) Vomiting/nausea (16, 22%) Vertigo (13, 17.5%) Seizure (12, 16%) Impaired vision (11, 15%) Paralysis (9, 12%)	Surg + RT + CHT (21, 28%) RT (20, 27%) Surg + RT (14, 19%) Surg (11, 15%) RT + CHT (6, 8%) Surg + CHT (2, 3%)	6.2
Chen [31] 2011	10	1.9%	Serous (7, 70%) MC (2, 20%) CC (1, 10%)	G2 (4, 40%) G3 (6, 60%)	II (1, 10%) III–IV (9, 90%)	56.6	24.3	1 (10%) vs. 9 (90%)	Extremity weakness (2, 20%) Blurred vision (2, 20%) Headache (3, 30%) Dizziness (2, 20%) Consciousness disturbance (1, 10%) Seizure (1, 10%)	RT + CHT (5, 50%) RT (3. 30%) Surg + RT (1, 10%) No treatment (1, 10%)	3
Cormio [32] 2011	20	5%	Serous (14, 70%) ED (3, 15%) Other (3, 15%)	G1 (1. 5%) G2 (4, 20%) G3 (15, 75%)	I (1, 5%) III (16, 80%) IV (3, 15%)	55.5	32.7	11 (55%) vs. 9 (45%)	NA	Surg + RT+ CHT (3, 15%) CHT + RT (3, 15%) Surg + CT (3, 15%) RT (3, 15%) No treatment (3, 15%) Surg (2, 10%) Surg + RT (2, 10%) CHT (1, 5%)	17.6
Chiang [33] 2011	64	1.2%	Serous (32, 50%) MC (5, 8%) ED (7, 11%) CC (11, 17%) Others (9, 14%)	G2 (11, 17%) G3 (33, 52%) NA (20, 31%)	I (8, 13%) II (4, 6%) III (36, 56%) IV (16, 25%)	52	22	18 (28%) vs. 46 (72%)	NA	RT + CHT (25, 39%) Surg + RT + CHT (12, 19%) RT (13, 20%) No treatment (7, 11%) Surg + RT (5, 8%) Surg (2, 3%)	8
Ogino [34] 2012	16	NA	Serous (4, 25%) CC (2, 12%) NA (8, 50%) Others (2, 12%)	NA	NA	53	27.5	6 (37%) vs. 10 (63%)	Extremity weakness (5, 31%) Seizure (2, 12%) Aphasia (1, 6%) Headache (1, 6%) Dysarthria (1, 6%) Dizziness (1, 6%)	RT (13, 81%) Surg + RT (3, 19%)	12.5
Nasu [35] 2013	56	NA	Serous (33, 59%) CC (7, 12%) ED (6, 11%) MC (2, 4%) Others (4, 7%) NA (4, 7%)	NA	I (7, 12%) II (1, 2%) III (32, 57%) IV (16, 29%)	55.8	25.2	30 (54%) vs. 26 (46%)	NA	RT (21, 37%) RT + CHT (11, 20%) Surg+ RT (8, 14%) No treatment (6, 11%) Surg + RT + CHT (5, 9%) CHT (3, 5%) Surg + CHT (1, 2%) Surg (1, 2%)	12.5
Niu [36] 2013	12	NA	Serous (10, 83%) Others (2, 17%)	G2 (1, 8%) G3 (11, 92%)	III (10, 83%) IV (2, 17%)	56	19	4 (33%) vs. 8 (77%)	NA	RT (4, 33%) RT + CHT (3, 25%) Surg + RT +CHT (3, 25%) Surg + RT (1, 8%) No treatment (1, 8%)	17
Teckie [37] 2013	60	NA	Serous (42, 70%) ED (8, 13%) CC (1, 2%) MC (1, 2%) Others (8, 13%)	G1 (2, 3%) G2 (7, 12%) G3 (49, 82%) NA (2, 3%)	I (3, 5%) II (4, 7%) III (40, 67%) IV (13, 21%)	56	41	28 (47%) vs. 32 (53%)	NA	RT (38, 63%) Surg + RT (22, 37%)	9.7
Gressel [38] 2015	19	NA	Serous (15, 79%) CC (2, 10,5%) NA (2, 10,5%)	NA	I (1, 5%) II (2, 11%) III (6, 32%) IV (10, 52%)	61	23	8 (42%) vs. 11 (58%)	NA	Surg + RT (9, 47%) RT (6, 32%) Surg (2, 10.5%) No treatment (2, 10.5%)	9
Gilani [39] 2016	13	NA	Serous (5, 39%) Others (5, 39%) NA (3, 23%)	NA	I (1, 8%) III (9, 69%) NA (3, 23%)	60	31	NA	NA	NA	23
Marchetti [40] 2016	174	NA	Serous (135, 77.6%), ED (13, 7.5%), CC (9, 5.2), MC (1, 0.6%), others (14, 8.1%) NA (2, 1.1%)	G1 (3, 1.7%), G2 (24, 13.8%) G3 (135, 77.6%), NA (12, 6.9%)	I–II (8, 4.6%) III (134, 77%), IV (32, 18.4%)	57	26	73 (42%) vs. 100 (57.4%) NA: 1 (0.6%)	Headache (58, 33.3%), Vertigo (40, 23%), Confusion (30, 17.2%), Nausea and vomiting (28, 16.1%), Seizures (17, 9.8%)	RT + CT (55, 31.6%), RT + CHT + Surg (41, 23.7%), RT (38, 21.8%), RT + surg (11, 6.3%) No treatment (11, 6.3%) CHT (8, 4.6%), CHT + surg (6, 3.4%), Surg (4, 2.3%),	12
Matsunaga [41] 2016	33	NA	Serous (23, 70%) MC (4, 12%) CC (3, 9%) ED (2, 6%) Others (1, 3%)	NA	NA	59	24	15 (45%) vs. 18 (55%)	NA	NA	8
Mittica [42] 2017	11	NA	Serous (9, 82%) ED (2. 18%)	G2 (1, 9%) G3 (10, 91%)	II (2, 18%) III (6. 55%) IV (3, 27%)	61	23	8 (73%) vs. 3 (27%)	Headache (2, 18%) Vertigo (2, 18%) Ataxia (2, 18%) Aphasia (1, 9%) Confusional state (1, 9%) NA (3, 27%)	Surg + RT (5, 45%) Surg + RT + CHT (3, 27%) Surg (2, 18%) Surg + CHT (1, 9%)	7
Seber [43] 2017	33	NA	NA	G2 (18, 55%) G3 (15, 45%)	III (24, 73%) IV (9, 27%)	57	22	15 (45%) vs. 18 (55%)	NA	CHT + Surg/RT (22, 67%) Surg +/- RT (19, 58%) Surg + RT + CHT (5, 4%) RT (2, 6%)	15
Kwon [44] 2018	56	2,8%	Serous (32, 57.2%) ED (5, 8.9%) Others 19 (33.9)	NA	I–II (6, 10.7%) III (19, 34%) IV (26, 46.4%) NA (5, 8.9%)	NA	27	24 (42.9%) vs. 32 (57.1%)	NA	RT (33, 58.9%) Surg (5, 9%) No treatment (10. 17.8%) Surg + RT (8. 14.3%)	NA
Wohl [45] 2019	25	NA	Serous 11 (73.3%)	NA	NA	58	42.3	18 (72%) vs. 7 (28%)	Headache (13, 53%) Motor deficit (8, 32%) Dysphasia (8, 32%) Seizure (3, 12%)	Surg + RT (11, 44%) RT (9, 36%) Surg (5, 20%)	35.8
Da Costa [46] 2019	26	4.6%	Serous (17, 65.4%) ED (2, 7.7%) Others (7, 26.8%)	NA	I–III (20, 76.9%) IV (5, 19.2%) NA (1, 3.8%)	NA	31.7	8 (30.7%) vs. 18 (69,3%)	NA	RT (13, 79.2%) Surg (8, 20.8%)	10.8
Keskin [47] 2019	21	NA	Serous (12, 57%) ED (1, 5%) CC (1, 5%) Others (7, 33%)	G2 3 (14)G3 18 (86)	I (1, 5%) III (17, 81%) IV (3, 14%)	NA	32	10 (48%) vs. 11 (52%)	NA	RT (8, 38%) RT + CHT (7, 34%) Surg (3, 14%) Surg + RT + CHT (3, 14%)	9
Mittica [48] 2020	29	NA	Serous (25, 86%) CC (2, 7%) Other (2, 7%)	G3 (29, 100%)	II (2, 7%) III (19, 65%) IV (8, 28%)	57	25	12 (41.4%) vs. 17 (58.6%)	Headache (10, 34%) Motor deficit (8, 28%) Seizures (3, 10%) Aphasia (3, 10%) Vomiting (2, 7%) Vertigo (2, 7%)	RT + CHT (7, 24%), Surg + RT (6, 21%) RT (6, 21%), RT + CHT + Surg (4, 14%) Surg (3, 10%), RT + surg (1, 3%) CHT + surg (1, 3%), No treatment (1, 3%)	12
Total	1135	1,34% (0.49%-6.1%)	Serous (682, 63.7%) ED (80, 7.5%) MC (62, 5.8%) CC (49, 4.6%) Other (182, 17%)	G1 (17, 2%) G2 (174, 21%) G3 (566, 69%)	I–II (118, 12%) III–IV (865, 86%)	55,9 (50.4–60.3)	27,3 (14.5–46)	450 (41,4%) vs. 632 (58, 1%)	Not Performed	Not Performed	10.1 (3–35.8)

OC: ovarian cancer, BM: brain metastasis, yrs: years, mo: months, NA: not available, ED: endometrioid, CC: clear cell, MC: mucinous, RT: radiotherapy, Surg: surgery, CHT: chemotherapy. The incidence of BMs was calculated only for studies in which the cohort of primary tumors was reported. The total numbers for ”histotype“, ”grade“ and ”FIGO Stage at OC diagnosis“ were provided only for studies in which these characteristics of primary tumors were reported.
